# Negative self-referential processing is associated with genetic variation in the serotonin transporter-linked polymorphic region (5-HTTLPR): Evidence from two independent studies

**DOI:** 10.1371/journal.pone.0198950

**Published:** 2018-06-13

**Authors:** Justin Dainer-Best, Seth G. Disner, John E. McGeary, Bethany J. Hamilton, Christopher G. Beevers

**Affiliations:** 1 Department of Psychology, The University of Texas at Austin, Austin, Texas, United States of America; 2 Institute for Mental Health Research, The University of Texas at Austin, Austin, Texas, United States of America; 3 Department of Psychiatry and Human Behavior, Brown University, Providence, Rhode Island, United States of America; 4 Providence Veterans Affairs Medical Center, Providence, Rhode Island, United States of America; Radboud Universiteit, NETHERLANDS

## Abstract

The current research examined whether carriers of the short 5-HTTLPR allele (in *SLC6A4*), who have been shown to selectively attend to negative information, exhibit a bias towards negative self-referent processing. The self-referent encoding task (SRET) was used to measure self-referential processing of positive and negative adjectives. Ratcliff’s diffusion model isolated and extracted decision-making components from SRET responses and reaction times. Across the initial (*N* = 183) and replication (*N* = 137) studies, results indicated that short 5-HTTLPR allele carriers more easily categorized negative adjectives as self-referential (i.e., higher drift rate). Further, drift rate was associated with recall of negative self-referential stimuli. Findings across both studies provide further evidence that genetic variation may contribute to the etiology of negatively biased processing of self-referent information. Large scale studies examining the genetic contributions to negative self-referent processing may be warranted.

## Introduction

Negatively biased information processing is known to be a risk factor for the development of future depression [1]. Negatively biased self-referent processing, in particular, has been shown to be strongly associated with depression risk [2]and is a central feature of contemporary cognitive models of depression [3, 4].

How one views the self is referred to as a cognitive schema—a representation of one’s past experiences that helps guide future categorization of self-referent information [4]. Cognitive schemas have been hypothesized to strongly influence the processing of incoming stimuli and can therefore influence whether positive or negative concepts are viewed as self-relevant [4]. An example of such processing includes easily associating negative terms (e.g., “useless”) with the self or, in contrast, difficulty associating positive terms (e.g., “lovable”) with the self. Further, negative schemas not only influence processing of incoming information, but they can also negatively bias other cognitive processes, such as recall of information. Thus, cognitive schemas and the processing of self-relevant information play an important role in the development of negative cognitive biases that, in turn, putatively confer risk for depression [5].

In his theoretical work, Beck has speculated that genetic factors may contribute to the development of negative cognitive schemas and biased self-referent processing [4]. One candidate gene that has been repeatedly linked to negative emotion-related phenotypes is the serotonin transporter [6]. The serotonin transporter (5-HTT) contributes to the active clearance of extracellular serotonin and appears to be influenced by a polymorphism in the proximal promoter region of the 5-HTT gene (i.e., the 5-HTT linked polymorphic region, or 5-HTTLPR; for a review, see [7]). The 5-HTTLPR is most commonly represented by two variants: a short (S) allele and a long (L) allele, although 5-HTTLPR expression may be influenced by an additional single-nucleotide polymorphism (SNP), namely, rs25531, which is composed of an adenine to guanine change at the sixth nucleotide in the first of two extra 20- to 23-base-pair repeats of the L allele [8]. The L allele with guanine at the sixth nucleotide (L_G_) and the S allele are similar in terms of transcriptional activity; therefore, only the L allele with adenine at the sixth nucleotide (L_A_) is associated with relatively increased transcriptional activity [9]. For the sake of brevity, we refer to the LG and S alleles as S′ and the LA allele as L′ throughout this article.

Past work has demonstrated that 5-HTTLPR variation is associated with other related cognitive biases, such as negatively biased attention [10, 11] and selective attention to negative stimuli [12]. A meta-analysis of ten studies (*N* = 807) found that carriers of the low expression variants of the 5-HTTLPR (SS, SL_G_, and L_G_L_G_) were more likely to display biased attention towards negative stimuli than high expression 5-HTTLPR genotypes (L_A_ homozygotes [12]). Differences in selective attention between 5-HTTLPR genotype groups were in the medium effect size range (which is very likely to be large over estimate of the true genetic effect).

More relevant to self-referential processing, prior work in a small sample of children found that S homozygotes recalled more negative words that were rated as self-descriptive following a negative mood induction than the other two genotype groups [13]. Even though it is now abundantly clear that complex traits, such as negative self-referential processing, are polygenic phenotypes (the so-called “fourth law” of behavioral genetics [14]), variation in the 5-HTTLPR appears to be somewhat consistently associated with negatively biased information processing. Thus, the 5-HTTLPR appears to be a reasonable genetic candidate to examine in the context of negative self-referential processing, with the caveat that it will only provide a very tentative and incomplete glimpse into the genetic architecture of self-referent processing of negative information.

The self-referent encoding task (SRET) [15] is often used to measure self-referent processing. The SRET is a two-choice, affective decision-making task that is typically paired with incidental recall of the presented stimuli. It involves participants making categorical decisions as to whether or not trait adjectives are self-descriptive. After a waiting period, participants are then asked to recall the adjectives presented during the SRET. Typically, depressed individuals endorse more negative adjectives as self-referential than non-depressed individuals [16]. This bias in self-referential processing is also present in individuals who have remitted depression [17], suggesting that negative cognitive biases persist even when symptoms are no longer evident. Furthermore, prior work has suggested that children at high-risk for depression display more negative processing on the SRET [18], suggesting that negative cognitive schemas predate the onset of depression.

Past research with the SRET has used the number of negative self-referent adjectives recalled as a proxy for negative self-schema (e.g., [17]). In the current study, we collected incidental recall but also supplemented it with a measure of decision-making bias by utilizing the diffusion model to analyze the reaction time and endorsement data ([19]; for its use in clinical research, see [20]). The diffusion model deconstructs reaction time for two-choice decision tasks into components that capture the encoding, decision-making, and motor response processes. Thus, a strong benefit of this approach is the ability to isolate and extract decision-making bias from non-decisional components (such as motor response) contained within reaction time responses [21].

**Fig 1:**
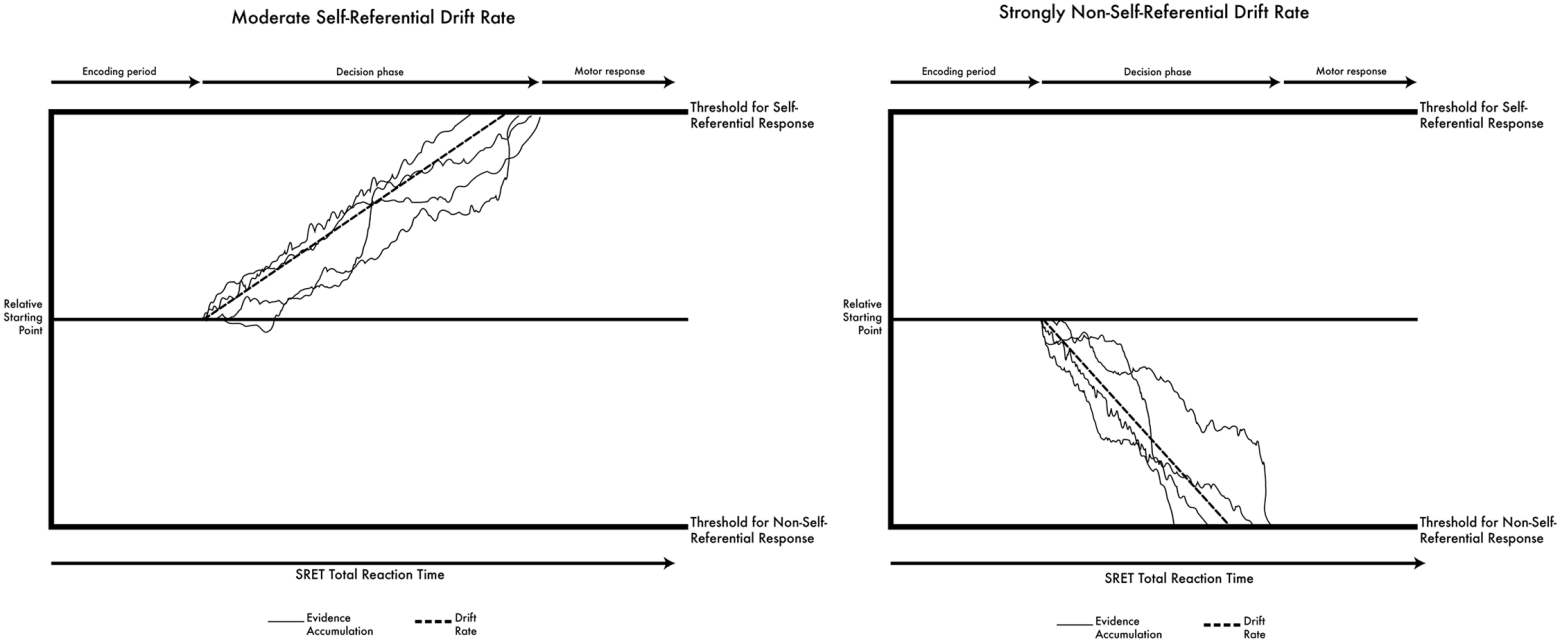
Illustration of the diffusion model. The two schematics shown here represent a subset of trials selected from hypothetical results for two conditions. The left image displays a moderate, self-referential drift rate (i.e. positive slope, low absolute value), and the right image displays a strong, non-self-referential drift rate (i.e. negative slope, high absolute value). Overall SRET reaction time is broken down into three components: encoding period, decision phase, and motor response. During the decision phase, evidence accumulation begins at the relative starting point (which varies across subjects) and continues until one of the thresholds is met. The time taken to reach the threshold across all trials is used to determine drift rate. Two drift rates were generated for each individual, one pertaining to decision making for positive adjectives and one for negative adjectives.

The diffusion model assumes that information is accumulated over the course of decision-making until a decision is reached [19] (see Fig 1) and that individuals differ in how efficiently people arrive at a decision. In diffusion model terms, drift rate is a parameter that quantifies the rate at which information is accumulated until a decision has been reached. Applied to the current study, drift rate provides a measure of the ease by which participants categorize each word as self-referential or non-self-referential. Drift rate can thereby be conceptualized as a measure of schema strength. For example, a positive drift rate (i.e., greater than 0) suggests that words are categorized as self-referent. The more positive the value, the easier it was for participants to decide that words were self-referent (a steeper positive slope in the left side of Fig 1). A negative drift rate (i.e., less than 0) suggests that words are categorized as not self-referent. The more negative the value, the easier it was for participants to decide that words were not self-referent (a steeper negative slope in the right side of Fig 1). Therefore, a drift rate for negative words that approaches or exceeds zero would be indicative of a strong negative schema, as this would indicate difficulty classifying negative words as not self-referent. Past research has shown strong connections between drift rate and depressive self-referent cognition [22, 23]. Although other components of the diffusion model are also associated with self-referent cognition (e.g., the relative starting point, thought of as indicating *a priori* bias in decision-making), they have not been as strongly associated with the negative self-referent processing that occurs in depression.

Given prior work documenting associations between the 5-HTTLPR and negatively biased processing, we hypothesized that carriers of the short allele would exhibit stronger self-referent processing biases for negative information—that is, they would more easily categorize negative adjectives as self-referential resulting in a larger (less negative) drift rate than individuals homozygous for the long 5-HTTLPR allele. We further hypothesized that negative self-referent processing would be associated with memory bias, such that a larger drift rate for negative words would be associated with greater recall of self-referential negative words.

Finally, given that the candidate gene literature is rife with non-replications [24], two studies were conducted: an initial study to test the main hypothesis, and, once a genetic association was observed, a replication study. Although a single, large study involving tens or hundreds of thousands of participants that allows for genome-wide testing would be ideal, demonstrating that a candidate genetic variant effect replicates in a second independent study is considered to be an important step towards reducing the likelihood of false positives in the literature [25]. Therefore, in the current work, Study 1 provides an initial test of the association between the 5-HTTLPR and self-referent processing, and Study 2 examined whether such an association could be replicated.

## Study 1

### Methods

#### Participants

One hundred and eighty-three participants aged 18–35 (106 female, 77 male; age *M* = 25.1, *SD* = 4.3) were recruited from the greater Austin community through fliers and newspaper advertisements. Participants were predominantly white (*n* = 111), Asian (*n* = 35), and Black (*n* = 7); those who did not provide racial background or identified as multiracial made up the remainder (*n* = 30). As a partial control for the risk of population stratification, we repeated analyses incorporating the 5-HTTLPR gene with only the white sample but did not see a substantively different pattern of results (see last section of Study 1 results). Twenty percent of participants (*n* = 36) across racial groups defined themselves as Hispanic.

Because we wanted to minimize potential psychiatric confounds, we ruled out anyone with diagnosable psychiatric disease. To do so, potential participants were screened via telephone using the Mini International Neuropsychiatric Interview (MINI), which screened for 17 different Axis I Diagnostic and Statistical Manual of Mental Disorders-IV (DSM-IV) disorders. The MINI has acceptable validity, test-retest, and inter-rater reliability [26, 27]. Participants who met criteria for a current or past psychiatric diagnosis (as determined by the MINI), were currently taking psychoactive medication, were currently in psychotherapy, or had a history of brain trauma were excluded from the study. Excluded participants were offered referrals to local mental health clinics.

Following genotyping described below, we conducted a power analysis to determine what power might result given a true effect size of Cohen’s *d* = 0.1, i.e., a small effect, and uneven groups (i.e., 34 participants with two L_A_ alleles and 149 with other genotypes). Given a two-tailed test with a significance level of .05, this study would achieve a power of 8.2%. This unfortunately is typical of candidate polymorphism studies, which are generally underpowered. We did not have the resources to conduct a fully powered study but instead recruited as many participants as possible for both studies.

#### Center for epidemiologic studies—depression scale (CESD)

The CESD [28] is a 20-item, self-report scale designed to assess the presence and severity of depressive symptoms over the past week. A total score of CESD <16 (not dysphoric) was required for study inclusion. This threshold is often used as a cut-off score to identify individuals at risk for MDD [28] and therefore is thought to reflect clinically significant levels of dysphoria. Participants completed CESD assessments via a secure online server. The CESD has been validated in community and psychiatric populations, and is considered highly reliable [28–30]. The CESD had strong reliability, Cronbach’s alpha = .86, 95% CI [.83, .88].

#### Genotyping

Genomic DNA was isolated from buccal cells and saliva using a modification of published methods [31–34]. Participants expectorated 2ml of saliva into a 50ml tube. Swabs previously impregnated and dried with lysis buffer (500*μl* of 1 MTris–HCl; pH 8.0) 500*μl* of 10% sodium docecyl sulfate; and 100*μl* of 5M sodium chloride were then added to the 50ml tube. Samples were stored at 4°C until the DNA was extracted.

The assay for 5-HTTLPR was a modification of that used by Lesch and colleagues [35]. The primer sequences are: forward, 5′-GGCGTTGCCGCTCTGAATGC-3′ (fluorescently labeled), and reverse, 5′-GAGGGACTGAGCTGGACAACCAC-3′ with yield products of 484 or 528 bp. To distinguish between the S, L_A_, and L_G_ fragments, the PCR fragment was digested with MspI by methods described in Wigg et al. [36]. Consistent with standard convention, the L_G_ fragment was treated as equivalent to S in all subsequent analyses [9, 37]. Thus, the L_A_L_A_ group consisted of individuals with two copies of the L_A_ allele, whereas the S′-carrier group consisted of individuals who carried the S or L_G_ allele. Two investigators independently scored allele sizes, and inconsistencies were reviewed and rerun when necessary. Results of an exact test for Hardy Weinberg proportions found no statistically significant deviation from Hardy Weinberg Equilibrium in the white subsample (*p* = .08; L_A_L_A_ = 23; L_A_L_G_ = 3; L_A_S = 40; L_G_L_G_ = 0; L_G_S = 2; SS = 43) or the entire sample (p = .08; L_A_L_A_ = 34; L_A_L_G_ = 3; L_A_S = 53; L_G_L_G_ = 0; L_G_S = 12; SS = 81).

#### Self-referent encoding task (SRET)

The SRET [15] is a computer-based task designed to assess schema strength by measuring the content and speed of the participants’ self-referential affective judgments. Participants saw a black screen with the word “Ready” displayed for 1000ms, followed by a word randomly selected from a list of 50 interpersonally oriented adjectives (25 positive and 25 negative) drawn from published norms and balanced for valence and arousal [38]. Participants were asked to decide as quickly as possible whether or not the word was self-descriptive. Trials were dropped if participants responded in fewer than 200ms, suggesting inattention or anticipatory guessing; an average of fewer than three trials were dropped, with no more than 10 total trials dropped for any individual participant. Following completion of a nonverbal decision task unrelated to the current study (i.e., a delay of 5-10 minutes), participants were then surprised with a recall task and given five minutes to recall as many of the SRET adjectives as possible. Primary outcomes were the drift rate for positive and negative adjectives identified by the diffusion model (discussed below) and number of self-referent word stimuli recalled.

Studies have shown that components of the SRET are reliably associated with depression symptomatology [23, 39]. The number of positive and negative endorsed words, as described above, show good test-retest reliability over two weeks, high levels of Cronbach’s alpha, and strong intra-task reliability [23]. Similar findings were evident for the drift rate for both positive and negative words [23]. In Study 1, for number of words endorsed, Cronbach’s alpha was equal to .85, 95% CI [.82, .88] for positive words and .87, 95% CI [.84, .89] for negative words.

#### Analysis

Ratcliff’s diffusion model [19, 20] was used to enhance the interpretability of the SRET reaction time data. The diffusion model is a sequential sampling technique designed to deconstruct reaction time for two-choice decision tasks into cognitive processing components separate from non-decision processes such as encoding and motor response [19]. The diffusion model assumes that, within each trial, decisions are formed through the accumulation of evidence until one of two response criteria has been met (e.g., for the SRET, whether a stimulus is self-referential or not self-referential). Once the threshold for that criterion has been met, the decision process concludes and a response is initiated. The diffusion model yields several parameters that describe characteristics of participant response. These include drift rate, relative starting point, threshold separation, response time constant, and differences in speed of response execution [19].

Of the diffusion model parameters, drift rate is most germane to the SRET, since it putatively measures the rate of evidence accumulation that leads to a decision about whether a stimulus is self-referent or not. For the SRET, a positive drift rate (i.e., drift >0) reflects evidence accumulation that leads to endorsing the stimulus as self-referential, whereas a negative drift rate (i.e., drift <0) reflects evidence accumulation that leads to rejecting the stimulus as self-referential (see Fig 1). The absolute value of drift rate reflects the efficiency of evidence accumulation, with larger absolute values reflecting a more efficient decision-making process. For example, a drift rate of 2 reflects more efficient endorsing of self-reference compared to drift rate of 1, while a drift rate of -2 reflects more efficient rejection of self-reference compared to a drift rate of -1. Negative and positive schema strength are operationalized as the drift rate for negative and positive adjectives, respectively. For people with a bias towards negative self-referent processing, we would expect a larger drift rate for negative adjectives (that is, they should inefficiently reject negative adjectives as self-descriptive or more efficiently endorse negative adjectives as self-descriptive). In contrast, for people with a bias towards positive self-referent processing, we would expect a smaller (more negative) drift rate, as they should efficiently endorse negative adjectives as not self-referent.

*Fast-dm* [40], a free software program, was used to implement the diffusion model. We used the Kolmogorov-Smirnov fitting method for optimizing parameters of the model, as suggested by Voss, Nagler, and Lerche [21]. This method is more robust in the presence of outliers, while still being effective at estimating stable parameters without requiring a large number of trials. Although other estimation methods have been suggested for use with small numbers of trials [41], past work has shown that the Kolmogorv-Smirnov estimation method achieves good results in the SRET [22] and that drift rates achieved using this estimation are strongly associated with depressive symptomatology [23]. (We include analyses with drift rates estimated by maximum likelihood methods in supplemental materials, S2 File.) Drift rate was estimated for positive and negative words trials separately. Following estimation, participants were excluded if their drift rates were above the median by more than four of a measure referred to as the double median absolute deviation (double MAD), a standard procedure intended to remove only extreme outliers. (One participant was dropped from this exclusion in Study 1, and six in Study 2.)

We used the package lme4 [42] and MASS [43] to fit statistical models in R (Version 3.4.3). As primary models involved repeated measure effects (positive and negative outcomes within participants), we used mixed effects linear models, with random intercepts of participant. Where *p*-values were reported, we compared nested models without the interaction, using likelihood ratio tests. Visualizations were created using ggplot2 [44]. Outliers were excluded before analysis as described above.

#### Procedure

Each participant was first screened for eligibility, and provided written informed consent to participate in the study. They took part individually, in a laboratory at the University of Texas at Austin, on a PC using E-Prime 2.0 (Psychology Software Tools) software to present stimuli and record reaction time data. Participants then provided saliva for genotyping under the direction of the experimenter. Upon completion of this and several tasks designed to measure cognitive functioning, not included in this report, participants were paid $8 per hour for their participation in the study. All procedures followed were in accordance with the ethical standards of the institutional review board at the University of Texas at Austin and the principles of the Declaration of Helsinki. The University of Texas at Austin’s Office of Research Support and Compliance provided approval for the original study (IRB #2011-10-0117).

### Results

#### Association between 5-HTTLPR and drift rate

Preliminary analyses first examined whether the short allele (S′: S and/or L_G_) homozygotes and heterozygotes did not differ from each other and therefore could be considered as a single genotype group (S′-carriers). A mixed effects regression analysis examined the association between 5-HTTLPR genotype (S′S′ and L_A_S′ only), stimuli valence (negative and positive), and the interaction between 5-HTTLPR and stimuli valence for the prediction of drift rate. The 5-HTTLPR × stimulus valence interaction was not significant, *β* = 0.23, 95% CI [-0.36, 0.81], *t* = 0.77, *p* = .44, indicating no significant differences between the S′ homozygotes and heterozygotes. As such, an S′-carrier group was formed and compared to an L_A_L_A_ group for remaining analyses.

**Fig 2:**
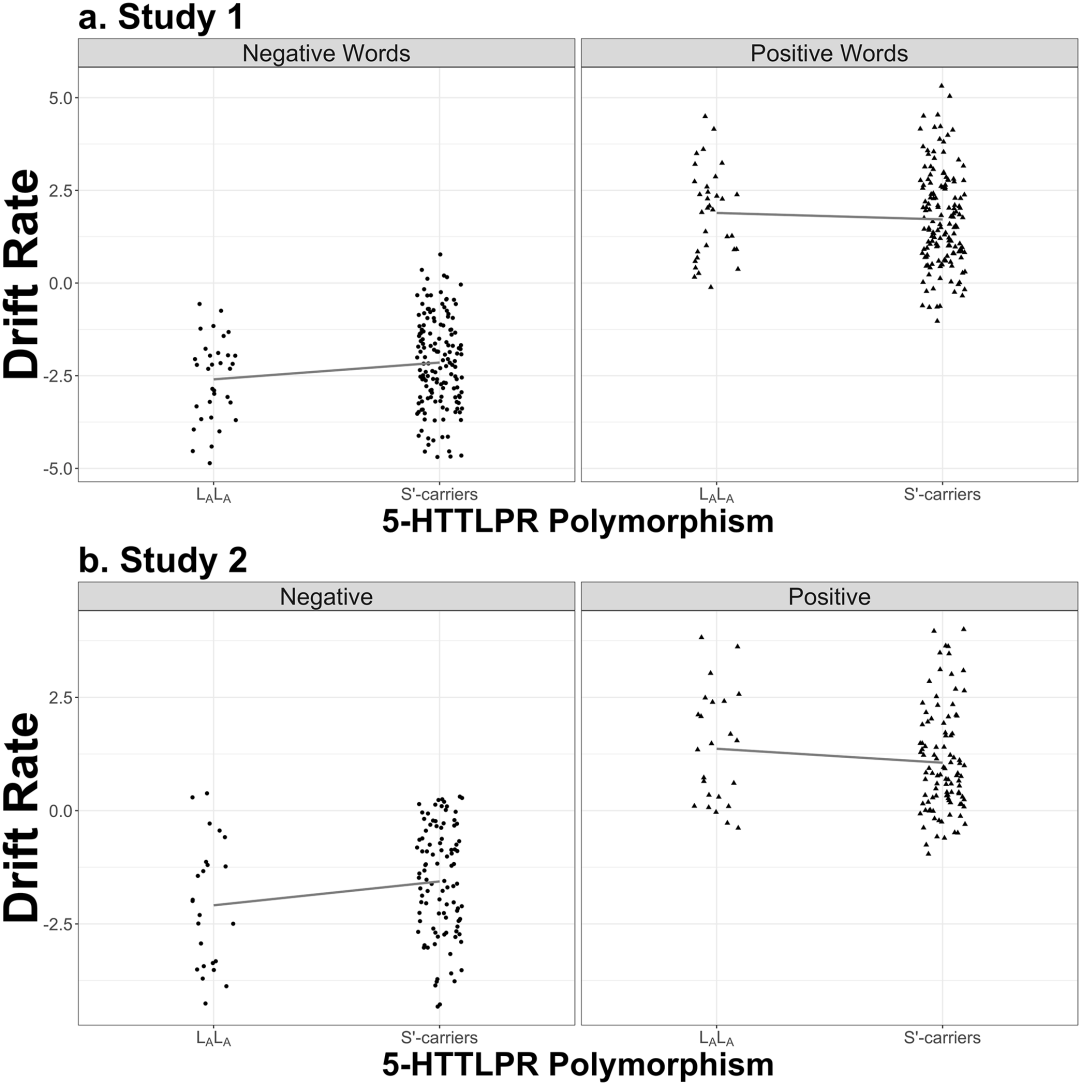
Association between 5-HTTLPR and drift rate for the self-referent encoding task. The top set of plots (a) show data from Study 1; the bottom set (b) show data from the replication, Study 2. The left plot shows the relationship between the 5-HTTLPR genotype and drift rate for negative adjectives and the right plot shows the relationship between the 5-HTTLPR genotype and drift rate for positive adjectives. The left plot shows that S′-carriers of the 5-HTTLPR polymorphism displayed a larger (less-negative) drift rate, indicating that they had more difficulty categorizing negative words as not self-referential. Points are jittered to so that all observations are presented.

The same analysis was carried out in all subjects examining the association between 5-HTTLPR genotype (L_A_L_A_ vs. S′-carriers), stimuli valence (negative and positive), and the interaction between 5-HTTLPR and stimuli valence. The resultant model’s *R*^2^ was .73, which had an *R*^2^ .003 better than the model without the interaction. The 5-HTTLPR × stimulus valence interaction neared significant, *β* = −0.63, 95% CI [-1.28, 0.02], *t* = −1.89, *p* = .06 (see Fig 2a). For positive stimuli, contrasts of marginal linear predictions indicated that L_A_L_A_ and S′-carriers did not differ in drift rate, *t*(180) = −0.72, *p* = .47, Cohen’s *d* = 0.14, 95% CI [-0.24, 0.52]. For negative stimuli, contrasts indicated that S′-carriers had significantly greater (i.e., less negative) drift rate than L_A_ homozygotes, *t*(180) = 2.00, *p* = .05, Cohen’s *d* = 0.38, 95% CI [0, 0.76]. S′-carriers were less efficient when deciding that negative stimuli were not self-referent than L_A_ homozygotes.

#### Association between drift rate and recall of self-referent word stimuli

**Fig 3:**
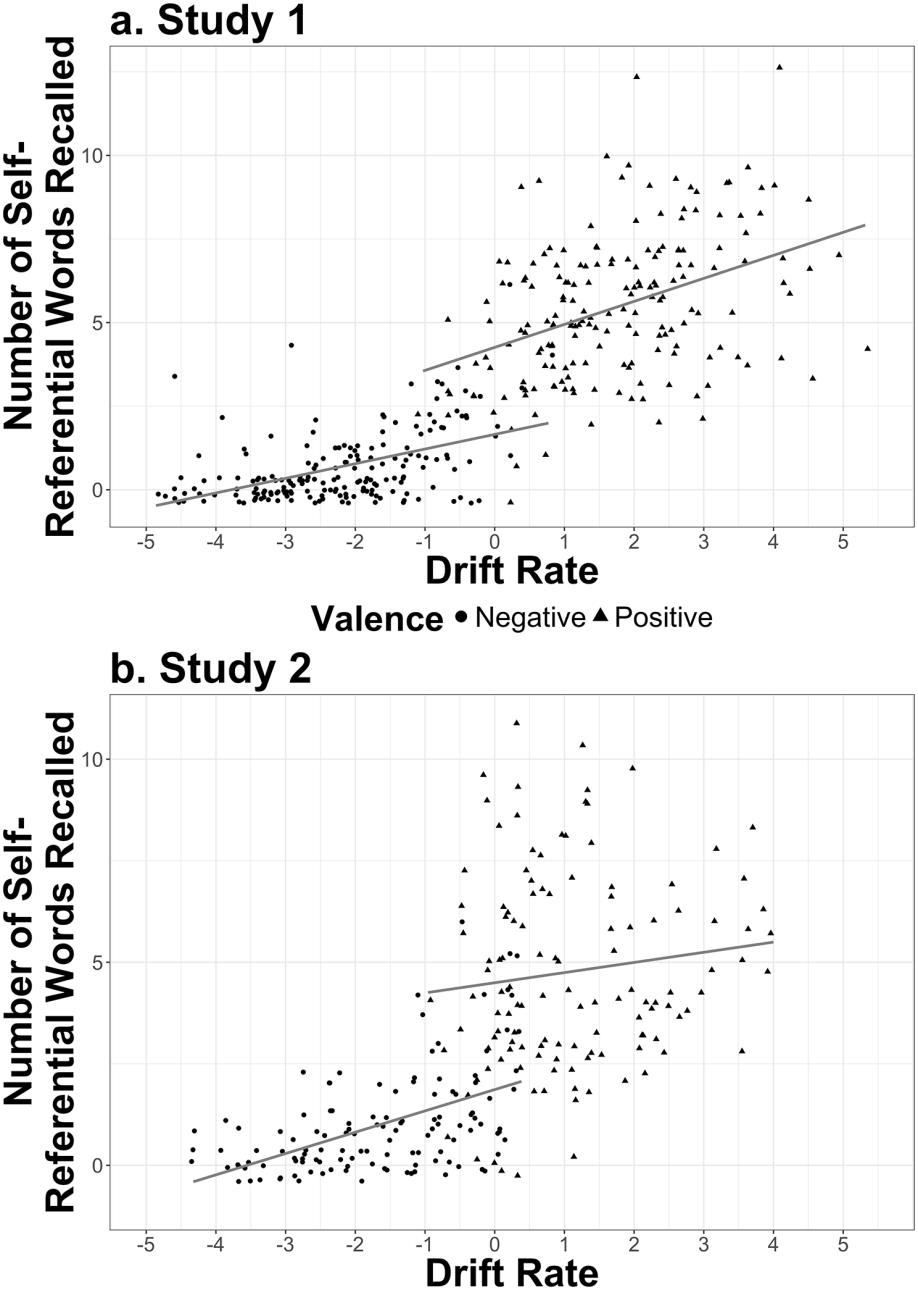
Association between drift rate and recall for self-referential words on the self-referent encoding task. The top plot (a) shows data from Study 1; the bottom plot (b) shows data from the replication, Study 2. The scatterplot shows the relationship between drift rate and recall of positive (circles) and negative (triangles) stimuli.

We ran a generalized mixed effects regression analysis with number of self-referent word stimuli recalled as the outcome variable and drift rate and stimuli valence as independent variables. Because stimuli recalled were not normally distributed, we used a negative binomial distribution to model the outcome, which adequately explained the combination of responses. The resultant model indicated a significant interaction between valence of word stimuli and drift rate, *β* = −0.56, 95% CI [-0.73, -0.39], *z* = −6.56, *p* < .001 (see Fig 3a). The model an *R*^2^ = .86, .06 better than the model without the interaction. These results suggest that as drift rate increased, so too did recall of self-referential stimuli. The slope of increase was steeper for negative (*β* = .68, 95% CI [0.52, 084], *z* = 8.32, *p* < .001) than positive stimuli (*β* = 0.12, 95% CI [0.07, 0.17], *z* = 5.00, *p* < .001). To examine whether the 5-HTTLPR genotype moderated these associations, analyses were repeated with 5-HTTLPR genotype added as an independent variable. The three-way interaction (drift rate × stimuli valence × 5-HTTLPR) was not significant, *β* = 0.10, 95% CI [-0.63, 0.43], *z* = −0.37, *p* = .71, which suggests no 5-HTTLPR moderation.

#### Recall of self-referent negative stimuli and valence

A mixed effects regression analysis with number of self-referent words recalled as the negative-binomial outcome variable and stimuli valence as the independent variable indicated a significant main effect for valence, *β* = 2.08, 95% CI [1.90, 2.27], *z* = 21.98, *p* < .001. The model had a strong fit, *R*^2^ = .79. Participants were much more likely to recall self-referent positive words (*M* = 5.46, *SD* = 2.15) than self-referent negative words (*M* = 0.68, *SD* = 1.04). To examine whether the 5-HTTLPR genotype moderated this association, analyses were repeated with 5-HTTLPR genotype added as an independent variable. The two-way interaction (valence × 5-HTTLPR) was not significant, *β* = −0.38, 95% CI [-0.89, 0.13], *z* = −1.47, *p* = .14, suggesting no 5-HTTLPR moderation.

#### 5-HTTLPR genotype differences for other diffusion model parameters

We examined 5-HTTLPR group differences for other diffusion model parameters (see Table 1). There were no significant differences in components between genotype groups. We compared the relative starting point both when allowed to vary between positive and negative words, and when set constant across both; neither showed significant differences between groups. Correlations between variables are available in supplementary materials S1 File.

**Table 1:** Means (SD) for depressive symptoms and diffusion model parameters presented as a function of 5-HTTLPR genotype.

	*Description*	Study 1				Study 2			
		*5-HTTLPR*		*t*	*p*	*5-HTTLPR*		*t*	*p*
		*S′-carr*	*L_A_L_A_*			*S′-carr*	*L_A_L_A_*		
		149	34			100	24		
CESD	Center for epidemiologic studies—depression scale	6.52 (4.25)	5.79 (4.54)	-0.84	.40	7.15 (3.85)	8.25 (4.06)	1.20	.24
Drift rate (*v*) for positive words	Rate of information accumulation, corollary of self-referent processing	1.72 (1.28)	-2.13 (1.22)	-1.36	0.17	1.06 (1.14)	1.37 (1.26)	-0.84	.03
Drift rate (*v*) for negative words	(*as above*)	1.80 (1.22)	-2.49 (1.32)			-1.56 (1.20)	-2.09 (1.38)		
Relative starting point (*zr*) for positive words	A priori bias in decision making. Values > .50 reflect bias towards self-reference.	0.65 (0.12)	0.64 (0.15)	0.90	.37	0.64 (0.15)	0.68 (0.13)	-1.86	.06
Relative starting point (*zr*) for negative words	(*as above*)	0.36 (0.13)	0.38 (0.17)			0.37 (0.13)	0.33 (0.15)		
Threshold separation (*a*)	Amount of information required for making a decision. Larger values reflect need for more information.	1.50 (0.53)	1.53 (0.44)	0.38	.71	1.66 (0.59)	1.61 (0.50)	-0.44	.66
Response time constant (*t*0)	Average duration of encoding and response execution (non-decisional processes).	0.61 (0.11)	0.6 (0.1)	-0.64	.52	0.61 (0.11)	0.61 (0.14)	0.008	.99
Differences in speed of response execution (*d*)	Larger values indicate response execution is faster for self-referent responses.	.01 (.04)	.02 (.03)	1.30	.20	0.02 (0.05)	0.02 (0.03)	-0.09	.93

#### Analyses with white participants only

Analyses were repeated with only white participants, as 5-HTTLPR genotype proportions may vary across a racially diverse sample. A mixed effects regression analysis examined the association between 5-HTTLPR genotype (L_A_L_A_ vs. S′-carriers), stimuli valence (negative and positive), and the interaction between 5-HTTLPR and stimuli valence for the prediction of drift rate. The trend was consistent with the whole sample, although the results were non-significant, *β* = −0.43, 95% CI [-1.16, 0.30], *t* = −1.13, *p* = .25. A generalized mixed effects regression analysis with number of self-referent word stimuli recalled as the negative binomial outcome variable, and drift rate and stimuli valence as independent variables, found a significant interaction, *β* = −0.59, 95% CI [-0.85, -0.34], *z* = −4.64, *p* < .001. However, there was no moderation by 5-HTTLPR genotype, *β* = −0.17, 95% CI [-0.80, 0.45], *z* = −0.54, *p* = .60. Similarly, a mixed effects regression with number of self-referent words recalled as the negative-binomial outcome variable and stimuli valence as the independent variable indicated a significant main effect for valence, *β* = 2.18, 95% CI [1.93, 2.43], *z* = 17.05, *p* < .001, but there was no moderation by 5-HTTLPR genotype, *β* = −0.13, 95% CI [-0.71, 0.45], *z* = −0.45, *p* = .65.

## Study 2: Replication

### Methods

#### Participants

Following completion and analysis of data from Study 1, an additional 137 participants were recruited through fliers and online advertisements from the Austin community to participate in a replication study (78 female, 52 male; age *M* = 23.0, *SD* = 4.5). To avoid confounding results due to differing proportions of 5-HTTLPR variation across a racially diverse sample, all participants in this replication study were white. Thirty-five identified as Hispanic or Latino. As in Study 1, participants were screened using the MINI over the phone, and did not meet criteria for any current mental illness or past MDD. All participants had CESD <16 at the time of participation. Only participants for whom genotyping succeeded were included in analyses.

Following genotyping, we conducted a power analysis to determine what power might result given a true effect size of Cohen’s *d* = 0.1, i.e., a small effect, and uneven groups (i.e., 26 participants with two L_A_ alleles and 111 with other genotypes). Given a two-tailed test with a significance level of .05, this study would achieve a power of 7.4%.

#### Procedure

An amendment to Study 1 was approved by the IRB at the University of Texas at Austin to permit replication data collection. Participants completed the CESD and SRET as described in Study 1. The CESD had strong reliability, Cronbach’s alpha = .88, 95% CI [.85, .9]. The SRET had one adjective replaced (“wicked” was replaced by “funny”, ensuring that 25 words remained in each condition). Number of words endorsed on the SRET continued to have strong reliability, Cronbach’s alpha = .87, 95% CI [.85, .9] for positive words and .85, 95% CI [.82, .87] for negative words. SRET trials were dropped in a similar manner as in Study 1 (i.e., if responses occurred in under 200ms), with no participants losing more than 10 trials.

Two salivary DNA collection methods were used in this study. For the first 75 participants, the same collection methods were used as described above. For the remaining sample, participants were asked to rub the inside of their cheeks and gums with cotton-tipped swabs, for 30s each, three times. The three swabs were stored in a 50ml tube containing the same lysis buffer as described in the previous collection method. Following swabbing, participants were asked to swish 10ml of distilled water in their mouths for 20s and then to expectorate into the tube. All tubes were stored in a locked refrigerator at 4°C until ready for extraction, where genomic DNA was extracted from buccal cells and saliva. All procedures were otherwise identical. Genotyping was carried out in the same laboratory. Results of an exact test for Hardy Weinberg proportions found no statistically significant deviation from Hardy Weinberg Equilibrium in the sample (*p* = .06; L_A_L_A_ = 26; L_A_L_G_ = 9; L_A_S = 58; L_G_L_G_ = 1; L_G_S = 9; SS = 34).

#### Analyses

As this was intended to replicate the original study, analyses were carried out exactly as before, using the same scripts, same software package for the diffusion model (i.e., *Fast-dm* [40]), same method of removing outlying drift rates, and same analysis steps. Six participants were dropped due to outlying drift rates using the same definition to identify outliers as in Study 1.

### Results

#### Association between 5-HTTLPR and drift rate

To determine whether the short allele (S′: S and/or L_G_) homozygotes and heterozygotes differed from each other, we used a mixed effects regression analysis examining the association between 5-HTTLPR genotype (for S′S′ and L_A_S′ only), stimuli valence (negative and positive), and the interaction between 5-HTTLPR and stimuli valence for the prediction of drift rate. The 5-HTTLPR × stimulus valence interaction was not significant, *β* = 0.10, 95% CI [-0.64, 0.85], *t* = 0.28, *p* = .78; there was no significant difference between the S′ homozygotes and heterozygotes. Thus, we again compared S′-carriers to an L_A_L_A_ group in further analyses.

We then examined the association between 5-HTTLPR genotype (L_A_L_A_ vs. S′-carriers), stimuli valence (negative vs. positive), and the interaction between the two. The 5-HTTLPR × stimulus valence interaction was significant, *β* = −0.84, 95% CI [-1.59, -0.09], *t* = −2.19, *p* = .03 and the model had *R*^2^ = .58, which was .008 better than the model without the interaction. Fig 2 shows that the replication data showed similar results to Study 1. For positive stimuli, contrasts of marginal linear predictions indicated that L_A_L_A_ and S′-carriers did not differ in drift rate, *t*(122) = −1.17, *p* = .24, Cohen’s *d* = 0.27, 95% CI [-0.19, 0.72]. Conversely, for negative stimuli, contrasts indicated a near significant effect of S′-carriers having greater (less negative) drift rate than L_A_ homozygotes, *t*(122) = 1.88, *p* = .06, Cohen’s *d* = 0.43, 95% CI [-0.03, 0.88] (see Fig 2b).

#### Association between drift rate and recall of self-referent word stimuli

We ran a generalized mixed effects regression analysis with number of self-referent word stimuli recalled as the outcome variable and drift rate and stimuli valence as independent variables. Because stimuli recalled were not normally distributed, we used a negative binomial distribution to model the outcome, which adequately explained the combination of responses. The resultant model indicated a significant interaction between valence of word stimuli and drift rate, *β* = −0.58, 95% CI [-0.78, -0.37], *z* = −5.57, *p* < .001. The model had an *R*^2^ = .75, .13 better than the model without the interaction. As drift rate increased, so too did recall of stimuli, *z* = 13.47, *p* < .001 (see Fig 3b). As in Study 1, the slope of increase was steeper for negative (*β* = 0.62, 95% CI [0.44, 0.81], *z* = 6.75, *p* < .001) than positive stimuli (*β* = .05, 95% CI [-0.03, 0.13], *z* = 1.28, *p* = .20). This slopes to compare are visible in Fig 3.

To examine whether the 5-HTTLPR genotype moderated these associations, this analysis was repeated with 5-HTTLPR genotype added as an independent variable. The three-way interaction (drift rate × stimuli valence × 5-HTTLPR) was not significant, *β* = 0.11, 95% CI [-0.41, 0.63], *z* = 0.42, *p* = .67, suggesting no 5-HTTLPR moderation.

#### Recall of self-referent negative stimuli and valence

A mixed effects regression analysis with number of self-referent words recalled as the negative-binomial outcome variable and stimuli valence as the independent variable indicated a significant main effect for valence, *β* = 1.57, 95% CI [1.43, 1.71], *z* = 22.41, *p* < .001, *R*^2^ = .55. Participants were much more likely to recall self-referent positive words (*M* = 4.77, *SD* = 2.5) than self-referent negative words (*M* = 0.99, *SD* = 1.26). To examine whether the 5-HTTLPR genotype moderated this association, analyses were repeated with 5-HTTLPR genotype added as an independent variable. The two-way interaction (valence × 5-HTTLPR) was not significant, *β* = −0.25, 95% CI [-0.61, 0.11], *z* = −1.36, *p* = .17, suggesting no 5-HTTLPR moderation.

#### 5-HTTLPR genotype differences for other diffusion model parameters

We examined 5-HTTLPR group differences for other diffusion model parameters (see Table 1). There was a marginally-significant difference in the relative starting point (*zr*), with the L_A_L_A_ group showing a weaker decisional bias towards self-referent outcomes for positive words, but not negative words, compared to the S′-carrier group. No other differences emerged between genotype groups. Correlations between variables are available in supplementary materials (S1 File).

### Combined sample

We combined the two samples, and conducted the analyses described above on the total sample. The total sample size was 306. First, we examined the association between 5-HTTLPR genotype (L_A_L_A_ vs. S′-carriers), stimuli valence (negative vs. positive), and the interaction between the two. The 5-HTTLPR × stimulus valence interaction was significant, *β* = −0.72, 95% CI [-1.22, -0.23], *t* = −2.85, *p* = .004. The model’s *R*^2^ was .67, .004 better than the model without the interaction. There was no significant difference between 5-HTTLPR groups in their response to positive words, *t*(304) = −1.16, *p* = .25, Cohen’s *d* = 0.17, 95% CI [-0.12, 0.45], but there was a significant difference between them in their response to negative words, *t*(304) = 2.88, *p* = .004, Cohen’s *d* = 0.42, 95% CI [0.13, 0.70].

A generalized mixed effects regression analysis with number of self-referent word stimuli recalled as the negative binomial outcome variable and drift rate and stimuli valence as independent variables found a marginally significant interaction, *β* = −0.10, 95% CI [-0.21, 0.004], *z* = −1.89, *p* = .06. There was a strong relationship indicating that increases in drift rate also increased self-referent recall of stimuli, *β* = 0.21, 95% CI [0.17, 0.24], *z* = 12.07, *p* < .001. The relationship was not moderated by 5-HTTLPR group, *β* = 0.03, 95% CI [-0.24, 0.30], *z* = .23, *p* = .82.

## Discussion

This study investigated the relationship between genetic variation in the serotonin transporter gene and negative self-referent processing. Findings from this study provide evidence that the presence of the short allele of the 5-HTTLPR polymorphism is associated with increased negative self-referent processing. Short 5-HTTLPR allele carriers had a larger drift rate for negative words, indicating greater difficulty categorizing negative words as not self-referential compared to long allele homozygotes. Drift rate for categorizing negative words, in turn, was associated with increased recall of self-referential negative stimuli. However, the 5-HTTLPR polymorphism did not moderate the connection between drift rate and memory bias for negative or positive stimuli.

Although findings from Study 1 were intriguing, we sought to determine whether the association between 5-HTTLPR variation and drift rate for negative stimuli observed in Study 1 could be replicated. Replication is particularly important in this area of research, as many initially observed candidate gene effects have not been subsequently replicated, casting doubt on the reliability of initial genetic discoveries [45]. Further, given that population stratification (i.e., presence of allele frequency differences between different subpopulations) and analytic flexibility can contribute to false discoveries [25], for the replication study we recruited a racially homogenous sample, implemented the identical research protocol, and followed an identical analytic plan using the same analysis scripts as in Study 1.

Results from the replication study were largely consistent with the initial study, and this consistency is demonstrated in the results for the combined samples reported below the replication. Most notably, we replicated a significant interaction between SRET stimulus valence and 5-HTTLPR genotype. As in Study 1, the S′ carrier group had a larger drift rate for negative stimuli compared to the L_A_L_A_ group and no genotype differences in drift rate were observed for positive stimuli. The genotype effect size for differences in drift rate for negative words in Study 1 was smaller (Cohen’s *d* = .38) than in Study 2 (Cohen’s *d* = .48), but both trends were in the same direction. Nevertheless, the effect size for the genetic effect in these studies is small, as indicated by the marginal increases in percentage variance explained by the addition of the interaction term in regressions; this is reasonable, as the true genetic contribution of a single genetic variant is likely to be a small portion of variance explained for a putatively polygenic complex trait like negative self-referential processing [14]. If the study sample sizes were much larger (*N*s in the thousands), we would predict that the 5-HTTLPR effect size for its association with drift rate would be more representative of the true effect size.

Nevertheless, the current findings provide further evidence for the connection between 5-HTTLPR variation and biased processing of negative information. An interesting literature is emerging which appears to link the 5-HTTLPR to a range of negative cognitive biases (for a review, see [12, 46–48], although one should keep in mind that publication bias is likely influencing this literature and meta-analyses likely overestimate the true association between the 5-HTTLPR and negative information processing biases. However, it is important to note that the 5-HTTLPR was not globally associated with negative cognitive bias in the current study, as there was no direct link with biased memory for affective stimuli in either study. These findings suggest that the 5-HTTLPR genotype may increase risk for depression by influencing how negative information about the self is processed. Such a bias, in turn, may facilitate how negative information is recalled, and work in concert with other factors to increase cognitive vulnerability to depression.

Although the current study found a genetic association with negative self-referent processing, it remains unclear to what degree negative cognitive biases are influenced by genetic variation. Because the heritability of these cognitive phenotypes is not known, it is unclear to what extent these small effect findings, such as the ones observed in the current studies, account for the cumulative genetic influence. Genetically informed studies using twin methodology indicate moderate heritability for self-reported negative cognitive biases, such as rumination [49] and attributional style [50]. There is also evidence from twin studies for a strong genetic contribution to basic cognitive processes that likely underlie negative cognitive bias, such as executive function [51–53] and memory [54].

In addition to twin studies, estimates of genetic contribution can also be obtained from unrelated individuals when genomewide genetic variation is measured directly. This approach, commonly known as SNP heritability, provides an estimate of how much variance in a phenotype can be explained by measured genetic variation. A large project recently examined the genetic basis of 17 intermediate psychophysiological phenotypes for psychiatric disorder in a general population sample [55]. Several of the phenotypes demonstrated moderate or greater SNP heritability (e.g., antisaccade eye tracking error, overall startle amplitude, P3 amplitude, alpha frequency), but several did not [56, 57]. We believe it would be enormously helpful to conduct similar studies on a larger scale with negative cognitive bias phenotypes, as such work would provide critical direction regarding which phenotypes for depression should be further examined for their genetic basis and which ones should not be pursued. However, no such study has been completed despite the strong research base supporting the role of negative cognitive bias in depression [5].

A notable feature of the current study was the use of the diffusion model to operationalize the decision-making component of self-referent processing. Such computational models provide an ability to access the decisional processes that are conceptualized to be the basis of schematic systems [20]. Precise measurements of cognitive phenomena are vital in order to find generalizable and reproducible results. Measurement error reduces precision in parameter estimates, which can be particularly problematic for small *N* studies and when hypothesized effects are expected to be relatively modest, as in many candidate gene studies [58]. Thus, while it is very important to use negative cognitive bias tasks with robust psychometric properties in general [59], this may be especially true for genetic association studies. Computational modeling of key cognitive processes may help with this and future research in this area should try to incorporate these approaches whenever possible.

There are several limitations to this research that are worth noting. First and most importantly, while we did include a replication study, both studies were underpowered—if the true effect were in fact quite small, we would have been relatively unlikely to find it with these sample sizes. Given the number of false positives that have been observed in psychiatric genetics, we would be more confident in our findings if we had demonstrated the observed association between 5-HTTLPR and negative self-referent processing in two large studies with thousands of participants—indeed, we would need at least 8,500 participants to collect data from sufficient numbers of L_A_L_A_ participants to achieve 80% power. We believe that this preliminary study supports the inclusion of self-referential processing as a phenotype in future large-scale genetic analysis of cognitive biases. However, as in other studies investigating candidate genes, there are likely other unmeasured variables that contribute to the putative genetic association, including variance in the linkage disequilibrium of measured variants with unmeasured variants, population stratification, and others. We also recognize that a significant limitation of this report is that we conducted multiple tests in these analyses, such that some of our statistical tests would not withstand a correction for multiple comparisons. There is also a possibility that 5-HTTLPR polymorphic variations are associated with the decreased depressive symptoms used as an entry criterion for this study; we cannot be certain without recruiting a depressed sample.

Although we tried to account for several confounding factors, such as current or past psychopathology, depression symptom severity, and race (in Study 2), this is ultimately a correlational study and the association between 5-HTTLPR variation and self-referent processing is also vulnerable to other, non-genetic, third variable explanations. Related to this point, the current study used a sample of healthy individuals, which is a common approach in this area of research [60]. Nevertheless, it will be important to conduct similar research in clinical samples to determine whether these results generalize to people who are currently depressed.

Future research that uses the diffusion model with the SRET may consider adding trials to the existing task structure. The diffusion model converged for all participants in the study with acceptable model fit; however, diffusion model parameters typically have greater precision as number of task trials increases (e.g., 200 trials; for more detail, see [21]). Further, some studies have used the self-referent encoding task only after priming participants with a sad mood induction [17]. A sad mood induction may potentially enhance negative self-referent processing in vulnerable populations; future work examining the contribution of genetic variation to self-referent processing may consider such mood manipulations.

Nevertheless, the current study provides new evidence that genetic factors may contribute to the etiology of negative self-referent processing, suggests a new approach to measuring negative self-referent processing using the well-established SRET task, and further reinforces the fruitful synergy that can emerge from combining cognitive and genetic models of psychopathology (cf. [61]). We believe studying depression vulnerability across levels of analysis (e.g., genetic, cognitive, behavioral, environmental) will foster the development of comprehensive models of depression vulnerability and may ultimately help us to better understand the etiology of this pernicious disorder.

## Supporting information

S1 File Correlations between variables of interest are provided in supplementary S1 File. Correlation tables show the relationship between positive drift rate, negative drift rate, CESD, and the number of negative and positive self-referential words recalled.
(PDF)

S2 File Analysis with maximum likelihood estimation for drift diffusion model. These analyses repeat the primary analyses of the manuscript using a different estimation method.
(PDF)
