# Supplementary material for: Negative self-referential processing is associated with genetic variation in the serotonin transporter-linked polymorphic region (5-HTTLPR): Evidence from two independent studies
Source: PLoS One. 2018 Jun 13;13(6):e0198950. doi: 10.1371/journal.pone.0198950 (PMC5999110; doi:10.1371/journal.pone.0198950)
Supplement: S1 File — Correlation tables show the relationship between positive drift rate, negative drift rate, CESD, and the number of negative and positive self-referential words recalled. (PDF) [file pone.0198950.s001.pdf]

S1. Correlation Tables

Dainer-Best, Disner, McGeary, Hamilton, & Beevers

2018

Correlation Tables

These are supplementary information including correlations between parts.

Study 1

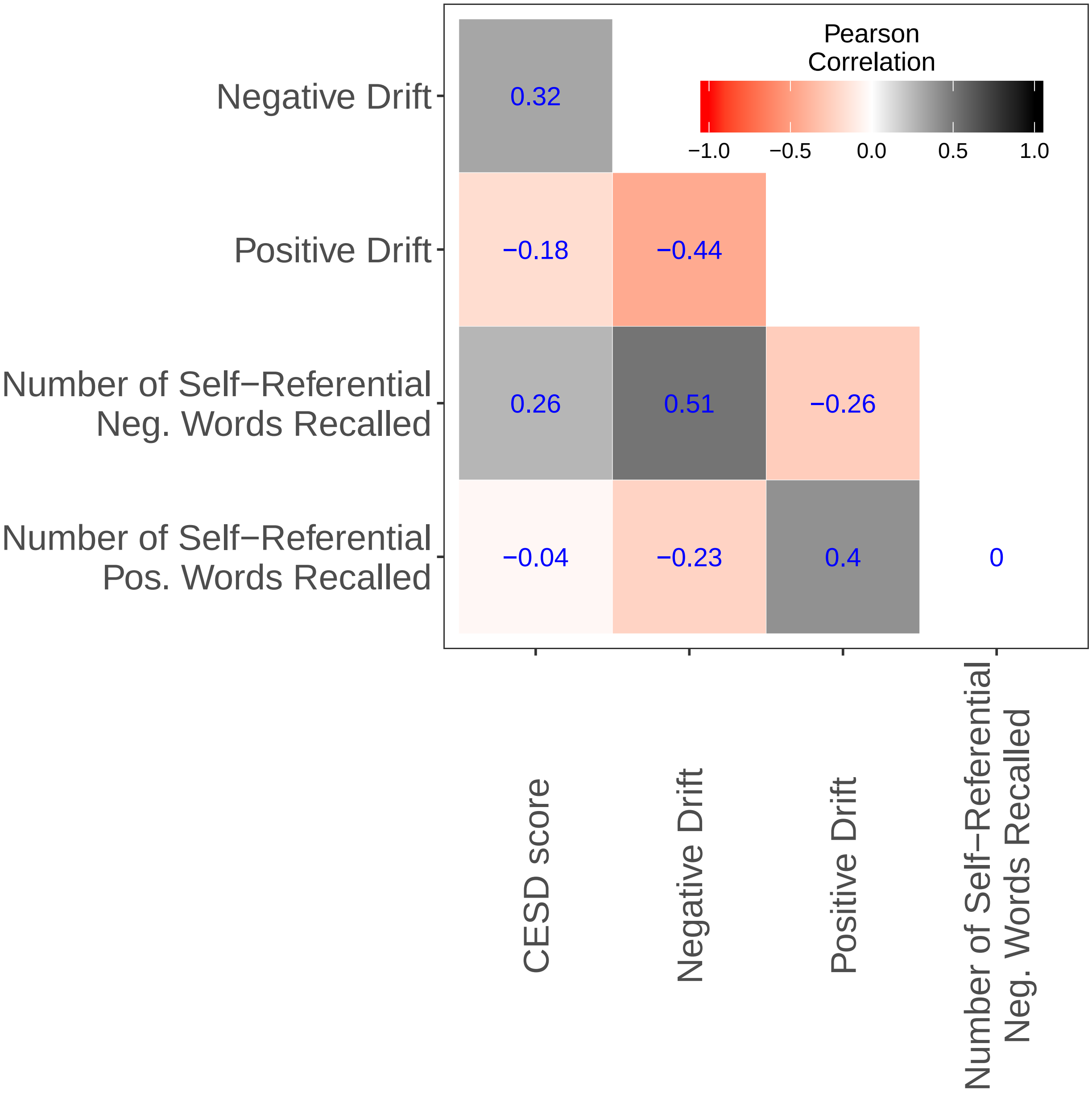

	CESD score	Neg. Drift	Pos. Drift	# Self-Referential Neg. Words Recalled
Neg. Drift	0.32**			
Pos. Drift	-0.18†	-0.44**		
# Self-Referential Neg. Words Recalled	0.26**	0.51**	-0.26**	
# Self-Referential Pos. Words Recalled	-0.04	-0.23*	0.4**	0

Table 1: ** < .001; * < .01; † < .05

Study 2

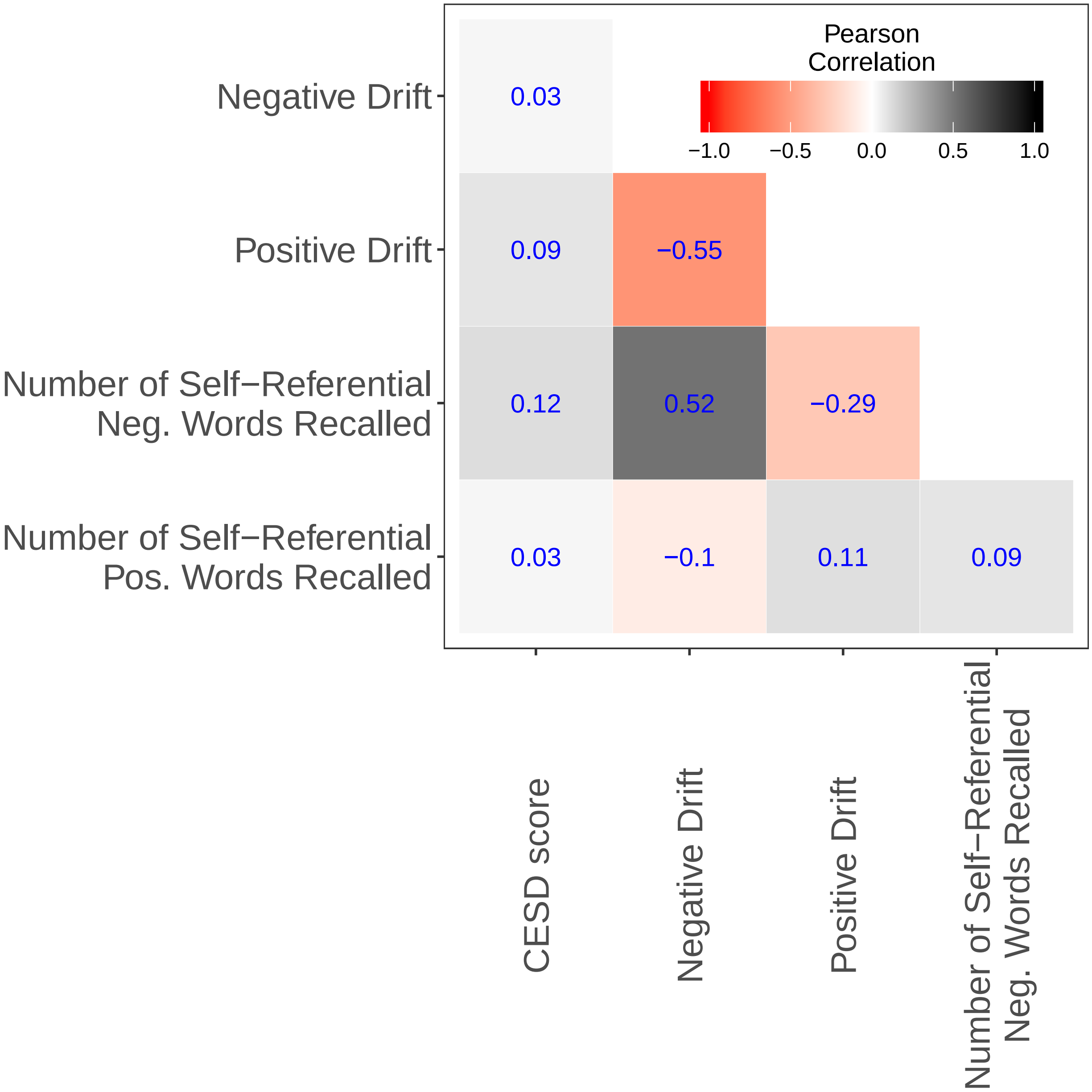

	CESD score	Neg. Drift	Pos. Drift	# Self-Referential Neg. Words Recalled
Negative Drift	0.03			
Positive Drift	0.09	-0.55**		
# Self-Referential Neg. Words Recalled	0.12	0.52**	-0.29*	
# Self-Referential Pos. Words Recalled	0.03	-0.1	0.11	0.09

Table 2: ** < .001; * < .01; † < .05
