# Supplementary material for: Negative self-referential processing is associated with genetic variation in the serotonin transporter-linked polymorphic region (5-HTTLPR): Evidence from two independent studies
Source: PLoS One. 2018 Jun 13;13(6):e0198950. doi: 10.1371/journal.pone.0198950 (PMC5999110; doi:10.1371/journal.pone.0198950)
Supplement: S2 File — These analyses repeat the primary analyses of the manuscript using a different estimation method. (PDF) [file pone.0198950.s002.pdf]

S2. Analysis with Maximum Likelihood Estimation for Drift Diffusion Model

Dainer-Best, Disner, McGeary, Hamilton, & Beevers

2018

Maximum likelihood estimation

As discussed in Voss, Voss, & Lerche, 2015, another potential method of estimating drift rate parameters is maximum likelihood estimation, which is based on an efficient algorithm that can be "especially useful in the case of small trial numbers" (p. 4), although it is also sensitive to outliers. The Kolmogorov-Smirnov estimation method used in the paper has been used in the past with the SRET, as we discuss, but we hoped to also test an estimation constrained as described by Voss, Voss, & Lerche for a small number of trials.

As such, we ran the diffusion model again using the updated *fast-dm* program (Voss, Voss, & Lerche, 2015), estimating based on the maximum likelihood estimation method and constraining parameters based on inter-trial variability to 0. We produce those results here.

Summary Statistics for Study 1

Some summary statistics about these drift rates. Voss & Voss (2007) and Voss, Voss, & Lerche (2015) both suggest that drift rate should fall within the range of -5 < drift < 5. That is not the case in these estimations, which we imagine are being pulled strongly by the clear distinction in responses from the SRET. The same results are seen below for the replication results.

	valence	participants	mean	sd	min	max
1	Negative	176	-20.62	18.66	-50.00	4.04
2	Positive	176	16.87	18.09	-1.32	50.00

Correlations for Study 1

	CESD score	Neg. Drift	Pos. Drift	# Self-Referential Neg. Words Recalled
Neg. Drift	0.04			
Pos. Drift	-0.02	-0.27**		
# Self-Referential Neg. Words Recalled	0.23*	0.04	0.08	
# Self-Referential Pos. Words Recalled	-0.01	-0.08	0.04	0.05

Table 1: ** < .001; * < .01; † < .05

These correlation tables indicate that the variables are not strongly associated with one another, nor the results of the diffusion model with depression symptomatology.

Primary Analyses for Study 1

We also conducted the two primary analyses from the paper using the estimates from maximum likelihood estimation described above.

Association between 5-HTTLPR and drift rate for Study 1

The first analysis examined the association between 5-HTTLPR genotype ($L_A L_A$ vs. S'-carriers), stimuli valence (negative and positive), and the interaction between 5-HTTLPR and stimuli valence.

Results indicated no interaction between genotype and valence in predicting drift rate.

```
## Linear mixed model fit by maximum likelihood  ['lmerMod']
## Formula: drift ~ gene * valence + (1 | id)
##    Data: melt.test
##
##      AIC      BIC   logLik deviance df.resid
##   3057.7   3080.8  -1522.8   3045.7      346
##
## Scaled residuals:
##     Min      1Q  Median      3Q     Max
## -1.6177 -0.7294 -0.2286  0.8361  1.8373
##
## Random effects:
##  Groups   Name        Variance Std.Dev.
##  id       (Intercept)   0.0     0.00
##  Residual             335.1    18.31
## Number of obs: 352, groups:  id, 176
##
## Fixed effects:
##                         Estimate Std. Error t value
## (Intercept)              -21.622      3.140  -6.887
## genes/x                    1.238      3.495   0.354
## valencePositive           40.604      4.440   9.145
## genes/x:valencePositive   -3.855      4.943  -0.780
##
## Correlation of Fixed Effects:
##             (Intr) gens/x vlncPs
## genes/x     -0.898
## valencePstv -0.707  0.635
## gns/x:vlncP  0.635 -0.707 -0.898
```

```
## Data: melt.test
## Models:
## fit.all.noi: drift ~ gene + valence + (1 | id)
## fit.all: drift ~ gene * valence + (1 | id)
##             Df    AIC    BIC  logLik deviance  Chisq Chi Df Pr(>Chisq)
## fit.all.noi  5 3056.3 3075.6 -1523.1   3046.3
## fit.all      6 3057.7 3080.8 -1522.8   3045.7 0.6077      1     0.4357
```

```
##                              2.5 %     97.5 %
## .sig01                          NA         NA
## .sigma                          NA         NA
## (Intercept)             -27.775383 -15.468388
## genes/x                  -5.613163   8.088213
## valencePositive          31.901699  49.306418
## genes/x:valencePositive -13.543367   5.833304
```

```
## R-squared for model: 0.51
```

```
## Delta R-squared: 8e-04
```

Association between drift rate and recall of self-referent word stimuli for Study 1

The second analysis used a generalized mixed effects regression analysis with number of self-referent word stimuli recalled as the outcome variable and drift rate and stimuli valence as independent variables. As in the paper, we used a GLM with a negative binomial distribution modeling the outcome variable of self-referent words recalled.

Again, although this model is statistically significant in the primary analysis, it does not appear so when drift rates are calculated using the maximum likelihood estimation.

```
## Generalized linear mixed model fit by maximum likelihood (Laplace
##   Approximation) [glmerMod]
##  Family: Negative Binomial(35677.41)  ( log )
## Formula: sr ~ valence * drift + (1 | id)
##    Data: sr.dat
##
##      AIC      BIC   logLik deviance df.resid
##   1181.5   1204.7   -584.8   1169.5      346
##
## Scaled residuals:
##     Min      1Q  Median      3Q     Max
## -2.3361 -0.8153 -0.2235  0.5473  6.3172
##
## Random effects:
##  Groups Name        Variance  Std.Dev.
##  id     (Intercept) 4.847e-14 2.202e-07
## Number of obs: 352, groups:  id, 176
##
## Fixed effects:
##                       Estimate Std. Error z value Pr(>|z|)
## (Intercept)           -0.363254   0.136758  -2.656   0.0079 **
## valencePositive        2.060561   0.143668  14.343   <2e-16 ***
## drift                  0.003549   0.005143   0.690   0.4902
## valencePositive:drift -0.002807   0.005438  -0.516   0.6057
## ---
## Signif. codes:  0 '***' 0.001 '**' 0.01 '*' 0.05 '.' 0.1 ' ' 1
##
## Correlation of Fixed Effects:
##             (Intr) vlncPs drift
## valencePstv -0.952
## drift        0.729 -0.694
## vlncPstv:dr -0.690  0.588 -0.946
## convergence code: 0
## Model failed to converge with max|grad| = 0.00137894 (tol = 0.001, component 1)
## Model is nearly unidentifiable: very large eigenvalue
##  - Rescale variables?
```

```
##                              2.5 %      97.5 %
## .sig01                          NA          NA
## (Intercept)           -0.631294362 -0.09521313
## valencePositive        1.778977183  2.34214525
## drift                 -0.006532134  0.01362941
## valencePositive:drift -0.013466419  0.00785146
```

```
## R-squared for model: 0.8
```

```
## Delta R-squared: 6e-04
```

Summary Statistics for Study 2

Some summary statistics about these drift rates for Study 2:

	valence	participants	mean	sd	min	max
1	Negative	129	-18.34	18.03	-50.00	1.23
2	Positive	129	15.76	17.92	-5.30	50.00

Correlations for Study 2

	CESD score	Neg. Drift	Pos. Drift	# Self-Referential Neg. Words Recalled
Neg. Drift	-0.04			
Pos. Drift	0	-0.38**		
# Self-Referential Neg. Words Recalled	0.11	0.01	0.02	
# Self-Referential Pos. Words Recalled	0.05	-0.05	0.04	0.09

Table 2: ** < .001; * < .01; † < .05

These correlation tables indicate that the variables are not strongly associated with one another, nor the results of the diffusion model with depression symptomatology.

Primary Analyses

We also conducted the two primary analyses from the paper using the estimates from maximum likelihood estimation described above.

Association between 5-HTTLPR and drift rate for Study 2

The first analysis examined the association between 5-HTTLPR genotype ($L_A L_A$ vs. S'-carriers), stimuli valence (negative and positive), and the interaction between 5-HTTLPR and stimuli valence.

Results indicated no interaction between genotype and valence in predicting drift rate.

```
## Linear mixed model fit by maximum likelihood  ['lmerMod']
## Formula: drift ~ gene * valence + (1 | id)
##    Data: replication.test
##
##      AIC      BIC   logLik deviance df.resid
##   2230.6   2251.9  -1109.3   2218.6      252
##
## Scaled residuals:
##     Min      1Q  Median      3Q     Max
## -2.0341 -0.7150 -0.1023  0.7984  2.0018
##
## Random effects:
##  Groups   Name        Variance Std.Dev.
##  id       (Intercept)   0.0     0.00
##  Residual             317.8    17.83
```

```
## Number of obs: 258, groups:  id, 129
##
## Fixed effects:
##                         Estimate Std. Error t value
## (Intercept)              -13.623      3.639  -3.744
## genes/x                   -5.793      4.033  -1.436
## valencePositive           27.936      5.146   5.428
## genes/x:valencePositive    7.572      5.704   1.327
##
## Correlation of Fixed Effects:
##             (Intr) gens/x vlncPs
## genes/x     -0.902
## valencePstv -0.707  0.638
## gns/x:vlncP  0.638 -0.707 -0.902
```

```
## Data: replication.test
## Models:
## fit.all.noi: drift ~ gene + valence + (1 | id)
## fit.all: drift ~ gene * valence + (1 | id)
##             Df    AIC    BIC  logLik deviance  Chisq Chi Df Pr(>Chisq)
## fit.all.noi  5 2230.4 2248.2 -1110.2   2220.4
## fit.all      6 2230.6 2251.9 -1109.3   2218.6 1.7562      1     0.1851
```

```
##                               2.5 %    97.5 %
## .sig01                           NA        NA
## .sigma                           NA        NA
## (Intercept)              -20.754832 -6.490268
## genes/x                  -13.698611  2.112366
## valencePositive           17.849606 38.022744
## genes/x:valencePositive   -3.607925 18.752172
```

```
## R-squared for model: 0.48
```

```
## Delta R-squared: 0.0036
```

Association between drift rate and recall of self-referent word stimuli for Study 2

The second analysis used a generalized mixed effects regression analysis with number of self-referent word stimuli recalled as the outcome variable and drift rate and stimuli valence as independent variables. As in the paper, we used a GLM with a negative binomial distribution modeling the outcome variable of self-referent words recalled.

Again, although this model is statistically significant in the primary analysis, it does not appear so when drift rates are calculated using the maximum likelihood estimation.

```
## Joining, by = "id"
```

```
## Generalized linear mixed model fit by maximum likelihood (Laplace
##   Approximation) [glmerMod]
##  Family: Negative Binomial(149.8807)  ( log )
## Formula: sr ~ valence * drift + (1 | id)
##    Data: maybe
##
##      AIC      BIC   logLik deviance df.resid
##    964.8    986.1   -476.4    952.8      252
##
## Scaled residuals:
```

```
##     Min      1Q  Median      3Q     Max 
## -1.9812 -0.9012 -0.1298  0.5777  3.8433 
## 
## Random effects:
##  Groups Name        Variance Std.Dev.
##  id     (Intercept) 0.06737  0.2596  
## Number of obs: 258, groups:  id, 129
## 
## Fixed effects:
##                         Estimate Std. Error z value Pr(>|z|)    
## (Intercept)           -5.713e-02  1.325e-01  -0.431    0.666    
## valencePositive        1.583e+00  1.411e-01  11.221   <2e-16 ***
## drift                  8.951e-04  5.157e-03   0.174    0.862    
## valencePositive:drift  8.914e-05  5.878e-03   0.015    0.988    
## ---
## Signif. codes:  0 '***' 0.001 '**' 0.01 '*' 0.05 '.' 0.1 ' ' 1
## 
## Correlation of Fixed Effects:
##             (Intr) vlncPs drift 
## valencePstv -0.893              
## drift        0.704 -0.650       
## vlncPstv:dr -0.631  0.454 -0.898
## convergence code: 0
## Model failed to converge with max|grad| = 0.00131718 (tol = 0.001, component 1)
## Model is nearly unidentifiable: very large eigenvalue
##  - Rescale variables?

##                              2.5 %     97.5 %
## .sig01                          NA         NA
## (Intercept)           -0.316837785 0.20257734
## valencePositive        1.306891886 1.86008238
## drift                 -0.009211716 0.01100189
## valencePositive:drift -0.011431375 0.01160965

## R-squared for model: 0.64

## Delta R-squared: -1e-04
```

Summary

The Kolmogorov-Smirnov (ks) method of estimating diffusion model parameters was associated with depression sytmptoms (CESD) and number of endorsed words (see S1), whereas the Maximum Likelihood (ml) method was not strongly associated with these metrics. Further, ml estimates were not “good” fits, as they were strongly affected by outliers in this estimation.

In terms of analyses discussed in the paper, these ml estimates failed to replicate the findings. We do not believe that these estimates model the data as well as the ks method, but we present these findings to provide a complete set of analyses.
